# Scald Injury-Induced T Cell Dysfunction Can Be Mitigated by Gr1^+^ Cell Depletion and Blockage of CD47/CD172a Signaling

**DOI:** 10.3389/fimmu.2020.00876

**Published:** 2020-05-08

**Authors:** Nadine Beckmann, Franziska Huber, Marc Hanschen, Barbara St. Pierre Schneider, Vanessa Nomellini, Charles C. Caldwell

**Affiliations:** ^1^Division of Research, Department of Surgery, University of Cincinnati, Cincinnati, OH, United States; ^2^Experimental Trauma Surgery, Klinikum Rechts der Isar, Technical University of Munich, Munich, Germany; ^3^Professor, School of Nursing, University of Nevada, Las Vegas, NV, United States; ^4^Division of Research, Shriner's Hospital for Children Cincinnati, Cincinnati, OH, United States; ^5^Division of Trauma Critical Care and Acute Care Surgery, Department of Surgery, University of Cincinnati College of Medicine, Cincinnati, OH, United States

**Keywords:** adaptive immunity, immunosuppression, myeloid-derived suppressor cells, immature reticulocytes, immune-checkpoint inhibitors

## Abstract

Infection is a common and severe complication of burn injury: Sepsis accounts for 47% of postburn mortality. Burn-induced T cell suppression likely contributes to the increased infection susceptibility in burn patients. However, little is known about the kinetics of T cell dysfunction after burn and its underlying mechanisms. In this study, we show in a murine scald injury model that T cell activation of both CD4^+^ and CD8^+^ T cells as well as T cell cytokine production is suppressed acutely and persistently for at least 11 days after burn injury. Purified T cells from scald-injured mice exhibit normal T cell functions, indicating an extrinsically mediated defect. We further show that T cell dysfunction after burn appears to be cell-to-cell contact dependent and can be ameliorated by depletion of myeloid-derived suppressor cells. These cells expand after burn injury, particularly a subset expressing the checkpoint inhibitor CD172a, and infiltrate germinal centers. Expression of CD172a appears to be driven by ingestion of immature reticulocytes. Immature reticulocytes are drastically increased in the spleen of scald mice and may contribute to immunosuppression through more direct mechanisms as well. Overall, our study newly identifies two cell populations, myeloid-derived suppressor cells and immature reticulocytes, as well as the CD47/CD172a-signaling pathways as mediators of T cell suppressors after burn and thus opens up new research opportunities in the search for new therapies to combat increased infection susceptibility and the associated morbidity and mortality in burn victims.

## Introduction

Infection is a common complication of burn injury, caused by skin barrier-loss and burn-induced immunosuppression. Despite the best prevention efforts, patients that do acquire an infection after burn injury suffer from increased morbidity and have a higher mortality risk. Investigators have previously determined that 47% of postburn mortality was the result of sepsis (1). To give burn victims their best chance of survival and provide them with the best possible care, a better understanding of burn-related immune dysfunction is urgently needed.

It is well-established that the early response to an infection is driven by innate immunity. Classically, the onset of the adaptive response was thought to occur after the innate response had subsided. However, research in the past decade has established a role of adaptive immunity in the acute response to an infection as well. In particular, T cells play a key role. T cell can enhance early innate function through production of IFN-γ and IL-17 (2). Additionally, regulatory and Th2 CD4^+^ T cells are known to mediate inflammation resolution (3).

T cells function and numbers are commonly decreased in critically ill burn patients (4–6). Indeed, decreases in IFN-γ, IL-2, and IL-12, and an increase in IL-4, IL-10, and IL-13 have been reported in both thermally injured patients and animal models (7–19). Further, it has been widely observed that following trauma, T cells undergo apoptosis (20–23). This could diminish TCR diversity through depletion of T cell clones and thus impair T cell effector functions against invading pathogens, which require a large repertoire of unique T cell receptors (TCRs). Additionally, apoptotic cells are capable of inhibiting the inflammatory program by releasing anti-inflammatory cytokines such as TGFβ and IL-10 (24, 25). They also release Annexin I, which attenuates IL-6 signaling and TNFα release from monocytes (26) and Lactoferrin, which inhibits neutrophil chemotaxis (27). In contrast to suppressing neutrophil recruitment, apoptotic cells induce the recruitment of macrophages, which clear apoptotic cells through phagocytosis (28). This triggers even more anti-inflammatory mechanisms, as it decreases macrophage production of GM-CSF, IL-1, IL-8, and TNFα, increases the production of TGFβ, PGE2, and PAF (29) and the release of pro-resolving lipid mediators, such as resolvins, protectins and maresin (30–32). Additionally, efferocytosis results in immune checkpoint expression: In sepsis studies, apoptosis of T cells has been linked to both increased expression of immune checkpoint inhibitor ligands (i.e., PD-L1) on myeloid cells, as well as their corresponding receptors on T cells (33–36). In addition to inducing T cell apoptosis, checkpoint inhibitor receptor/ligand interactions contribute to immunosuppression by inducing T cell exhaustion, which is characterized by a loss of T cell effector functions and decreased proliferation (37).

Targeting these changes could be a promising therapeutic approach to fight susceptibility to infections in burn patients. Data from septic patients show that patients whose TCR diversity remains low are at an increased risk for nosocomial infections and death (38) and in experimental sepsis models, the use of anti-apoptotic cytokine IL-7 improved survival, restored IFN-γ production and improved immune effector cell recruitment to the site of infection (39). In sepsis trials, the blockage of inhibitory immune checkpoints showed therapeutic efficacy (40, 41). More recently, anti-PD-L1 therapy was also shown to be effective in a murine model of scald injury and bacterial infection (42).

A novel concept of T cell suppression is immune-modulation by immature reticulocytes. Immature reticulocytes are precursors of red blood cells (RBCs). While they are normally generated in the bone marrow, stress conditions can induce extramedullary hematopoiesis, resulting in the presence of immature reticulocytes in the periphery (43). Immature reticulocytes express the erythroid lineage marker CD235 (human)/Ter119 (murine), transferrin receptor (CD71), VLA-4 (CD49d) and have residual RNA. The expression/presence of the last three decreases with increasing maturation (44–46). Immature reticulocytes were first attributed immunosuppressive properties by hampering immune cell activation and cytokine production through arginase 2 in a study using newborn mice, expanding the old concept that neonatal infection susceptibility is solely based on an underdeveloped immune system (44). In a newborn bacterial infection model, immature reticulocytes inhibited phagocytosis of *Bordetella pertussis* and their depletion with an anti-CD71 antibody significantly increased IFN-γ, IL-17 and anti-*B. pertussis*-antibody production (47). Immature reticulocytes have also been reported to express various immune checkpoint inhibitors (48–50). In co-incubation experiments, purified immature reticulocytes altered CD8^+^ T cell activation, inhibited conventional CD4^+^ and CD8^+^ T cell expansion, suppressed expansion of CD8^+^ regulatory cells, regulated cytokine responses to microbial products by myeloid cells and indirectly modulated T cell cytokine responses (49). Immature reticulocytes have also been observed in adults with hepatocellular carcinoma, which was associated with splenomegaly and promoted tumor progression through artemin production (51). In another tumor model, CD45^+^ Ter119^+^ CD71^+^ cells were identified as robust immunosuppressors (52). The study did not address why these cells express both the myeloid marker CD45 and the erythroid marker Ter119, but does show that the transcriptome of these cells closely resembles that of myeloid-derived suppressor cells (52). A possible explanation is that this population represents myeloid cells that ingested immature reticulocytes. Another interesting observation of the study is that these tumor-bearing mice were anemic, despite the increase in immature reticulocytes, indicating a blockage of erythrocyte maturation. Burn injury is known to cause anemia as well (53), however, the occurrence of immature reticulocytes after burn injury has not been investigated in detail yet.

The aim of this study was to determine the functionality of T cells in a time course after scald injury, since little is known so far about the duration of burn-induced T cell dysfunction. We also aimed to gain further mechanistic insights into burn-induced T cell dysfunction with regard to the mediating cell types and pathways involved, in the hope of identifying novel therapeutic targets to mitigate infection susceptibility after burn injury.

## Materials and Methods

### Mice

Male CD1 IGS mice were obtained from Charles River laboratories (Wilmington, MA) at 5 weeks of age and allowed to acclimate for 1 week prior to conducting experiments. All mice were housed in standard environmental conditions with *ad-libitum* access to pellet diet and water. All experiments were conducted between 8 and 11 a.m. using protocols approved by the Institution of Animal Care and Use Committee of the University of Cincinnati (IACUC number 08-09-19-01).

### Scald Burn Injury

We used a scald burn model as previously described (54). Briefly, 6-week old mice were randomized into two groups: scald and control. All mice were anesthetized with 4.5% isofluorane in oxygen. The back of the mice was shaven prior to placing them in a template exposing their dorsal surface, corresponding to 28% of their total body surface area (calculation based on the Meeh formula (55)). Scald mice were immersed in 90°C water for 9 s, yielding a full thickness, third degree, insensate legion. Control mice were immersed in room-temperature water instead. All mice were subsequently resuscitated intraperitoneally with 1.5 mL sterile normal saline. After the procedure, mice were allowed to recover on a 42°C heating pad for 3 h and subsequently returned to their home cage. Mice were monitored for any complications twice daily for the duration of the entire experiment.

### T Cell Re-stimulation

Mice were sacrificed by CO_2_ exposure and subsequent cervical dislocation on the indicated days after scald injury. Spleens were removed and splenocytes were isolated in RPMI medium (Lonza, Basel Switzerland) by gently mashing them through 70 μm filters (Corning, Corning, NY). Cell numbers were determined on a hemocytometer (Beckman Coulter, Brea, CA) and cells seeded at a density of 2 Mio cells/mL in 48-well tissue culture plates. Samples were stimulated with anti-CD3/CD28 coated Dynabeads (ThermoFisher, Waltham, MS) at a 1:1 ratio of beads to cells. Samples were incubated for 24 h or 48 h prior to assessment of T cell activation by flow cytometry. When indicated, 2 μg/mL anti-CD172a (clone P84, BioLegend, San Diego, CA) or 2 μg/mL anti-CD47 (clone miap301, BioLegend) were added for the duration of the stimulation.

### Flow Cytometry Analysis

Cells were isolated and treated as described for the respective experiment and analysis of cell surface antigen expression was performed. For intracellular staining, cells were fixed with 1% paraformaldehyde and permeabilized with 0.1% saponin. The following fluorescent-labeled antibodies were used: CD4 (clone RM4-5), CD8 (53-6.7), CD11b (clone M1/70), CD25 (clone PC-61), CD44 (IM7), CD45 (clone 30-F11), CD62L (clone MEL-14), CD69 (clone H1.2F3), CD155 (clone 3F1), CD172a (clone P84), CD200 (clone OX-90), CD273 (clone TY25), CD274 (clone MIH5), CD71 (clone RI7217), Gr1 (clone RB6-8C5), Ly6G (clone 1A8), Ter119 (clone TER-119) (all BioLegend or BD Bioscience, Franklin Lakes, NJ). Flow cytometry acquisition and analysis were performed on an Attune Flow Cytometer (Life Technologies, Foster City, CA).

### Cytokine Analysis

The IL-2 ELISPOT (CTL, Cleveland, OH) was conducted according to manufacturer's instructions. 30,000 cells/well were seeded and stimulated with anti-CD3/CD28 Dynabeads at a 1:1 ratio of beads to cells. IL-2 and IFN-γ concentrations in supernatants of the splenocyte cultures were quantified by cytometric bead assay (BD Bioscience) according to the manufacturer's instructions as previously described (56).

### Cell Purification

T cells were purified from spleens by magnetic bead separation using anti-CD90.2 microbeads (Miltenyi Biotec, Bergisch Gladbach, Germany) on an autoMACS separator (Miltenyi Biotec) according to the manufacturer's instructions. Similarly, Ter119^+^ cells were purified using the same system and anti-Ter119 microbeads (Miltenyi Biotec). CD71^+^ cells were isolated by jet-in-air cell sorting using an iSort Automated Cell Sorter (ThermoFischer) after staining with AF488-labeled anti-CD71 (clone RI7217, BioLegend).

### *In vivo* Gr1^+^ Cell Depletion

Mice were injected with 100 μg anti-Gr1 (clone RB6-8C5, BioXCell, West Lebanon, NH) in 100 μL sterile PBS *i.p*. on day 6 after scald injury. Spleens were harvested and neutrophil depletion confirmed by flow cytometry.

### Histology

Mice were sacrificed at the indicated times. Spleens were harvested, fixed in 10% formalin for 48 h, dehydrated in 70% ethanol for 72 h and embedded in paraffin. 5–10 μm thick sections were prepared and stained with hematoxylin and eosin. For detection of Gr1^+^ cells, sections were incubated with a primary antibody against anti-Gr1 (clone RB6-8C5, Bio-Rad, Hercules, CA), followed by horseradish-labeled secondary antibody and DAB. An isotype control was used to ensure specificity of the Gr1 staining. Samples were imaged on a Zeiss microscope. Invasion of Gr1^+^ cells into germinal centers was quantified by a blinded, experienced investigator.

### Statistics

Graphs depict each replicate in addition to the mean ± SEM. ROUT method was used to test for statistically significant outliers and data points were removed when the criteria were met (false discovery rate Q = 1%). Two groups were compared by Student *t*-test for unpaired samples or Wilcoxon signed-rank test for paired observations. Selective comparisons of three or more groups were calculated by ANOVA with the indicated *posttest*. A *p* ≤ 0.05 was considered significant.

## Results

### Scald Injury Persistently Impairs T Cell Activation

**Figure 1:**
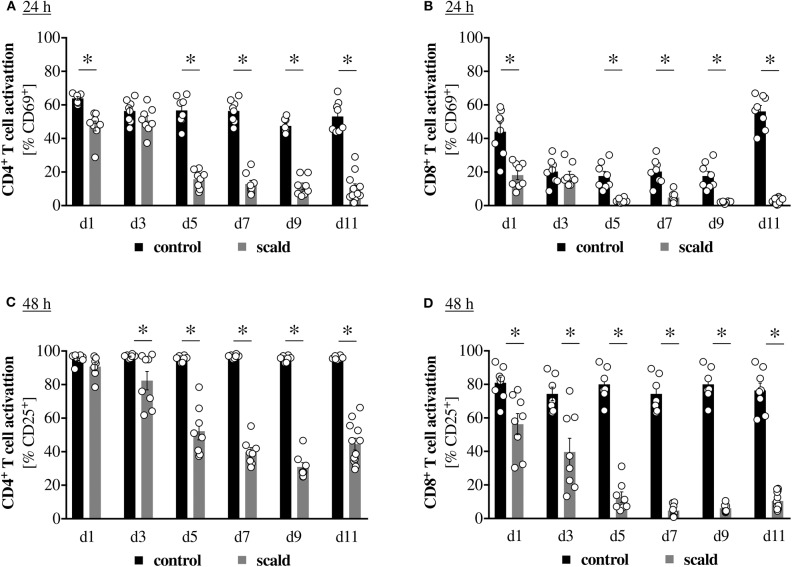
Scald injury persistently impairs T cell activation. CD1 outbred mice were subjected to scald injury (28% total body surface area (TBSA), full thickness). Controls did not undergo the procedure. Spleens were harvested on the indicated days after injury and splenocytes cultured for 24 h **(A,B)** or 48 h **(C,D)** in the presence of anti-CD3/CD28 coated beads. CD4^+^ **(A,C)** and CD8^+^ **(B,D)** T cell activation was determined by CD69 **(A,B)** and CD25 **(C,D)** positivity by flow cytometry. Graphs depict means ± SEM from two independent experiments in addition to each replicate. *n* = 8 (d1–d9), *n* = 12 (d11). Outliers were removed when permissible by ROUT method. *p < 0.05 compared to respective control (one-way ANOVA with Sidak posttest).

It is established that burn injury causes T cell dysfunction, but it is currently unclear how long the defect persists. To address this question, we conducted a temporal study. Mice were subjected to sham- or scald-injury, splenocytes were harvested every other day until Post Burn Day 11 (PBD11) and stimulated with anti-CD3/CD28 coated beads *ex vivo*. Early T cell activation, as indicated by CD69 positivity at 24 h, was persistently impaired on both CD4^+^ and CD8^+^ T cells with the exception of PBD3 (Figures 1A,B). Similarly, late T cell activation, as indicated by CD25 positivity at 48 h, was persistently decreased as well, starting on PBD3 in CD4^+^ T cells and on PBD1 in CD8^+^ cells. Neither parameter showed a clear indication of a beginning recovery on PBD11. We also analyzed T cell apoptosis upon *ex vivo* stimulation, but did not detect significant differences between controls and scalds at any timepoint (data not shown).

### Scald Injury Persistently Impairs Cytokine Production

**Figure 2:**
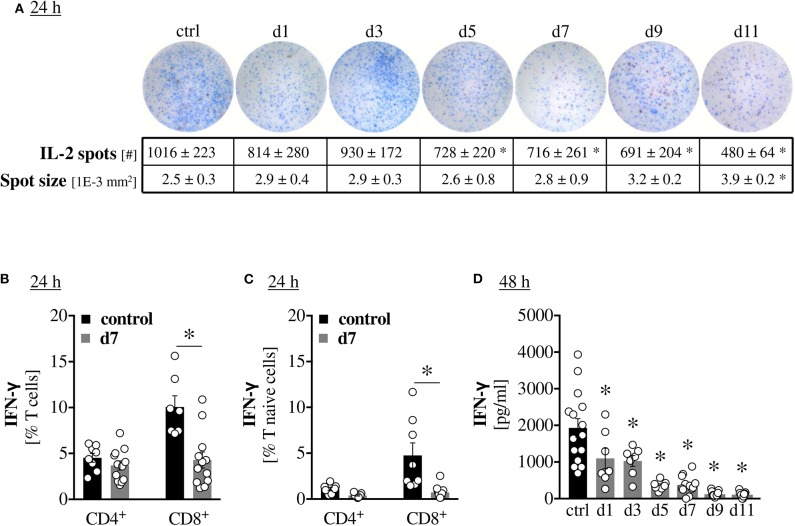
Scald injury persistently impairs cytokine production. CD1 outbred mice were subjected to scald injury (28% TBSA, full thickness). Controls did not undergo the procedure (ctrl). Spleens were harvested on the indicated days after injury and splenocytes cultured for 24 h **(A–C)** or 48 h **(D)** in the presence of anti-CD3/CD28 coated beads. IL-2 production was assessed by ELISPOT **(A)** The percentages of total and naïve T cells producing IFN-γ were determined by intracellular staining and flow cytometry **(B,C)**. IFN-γ production was quantified by cytometric bead assay **(D)**. Graphs depict means ± SEM from two independent experiments in addition to each replicate. *n* = 8 (controls), *n* = 12 (scald). For Figure 2D, the controls from each time-point were not significantly different from one another and were pooled for better legibility. Outliers were removed when permissible by ROUT method. *p < 0.05 compared to control (one-way ANOVA with Sidak **(B,C)** or Dunnett **(A,D)** posttest).

To determine whether impaired T cell activation is also accompanied by reduced functionality, we assessed cytokine production in a similar temporal manner. Fewer splenocytes from scald injured mice were able to produce IL-2 upon *ex vivo* stimulation. The defect became significant by PBD5 and persisted until PBD11 (Figure 2A). Production of IFN-γ by T cells was elucidated on PBD7 and scald mice showed a reduced percentage of total CD8^+^ cells and particularly naïve CD8^+^ cells producing IFN-γ upon *ex vivo* stimulation (Figures 2B,C). Quantification of IFN-γ production by the *ex vivo* splenocyte cultures showed a significant reduction starting on PBD1 and persisting until PBD11 with no indication of a beginning recovery.

### T Cell Purification Mitigates Scald-Induced T Cell Dysfunction

**Figure 3:**
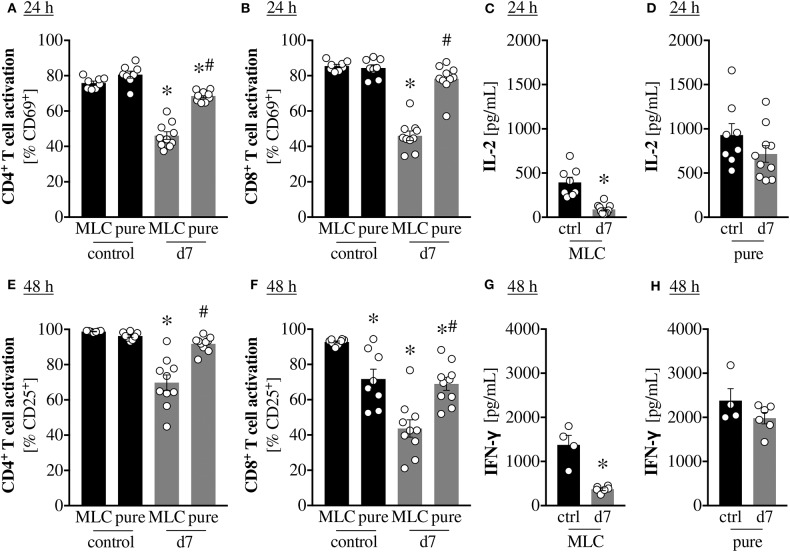
T cell purification mitigates scald-induced T cell dysfunction. CD1 outbred mice were subjected to scald injury (28% TBSA, full thickness). Controls did not undergo the procedure (ctrl). Spleens were harvested on day 7 after injury (d7), splenocytes isolated and CD90.2 positive cells purified by magnetic bead isolation. These purified cells (mostly T cells, “pure”) were cultured in the presence of anti-CD3/CD28 coated beads. As a control, splenocytes from the same mice were cultured without any purification (mixed leukocyte cultures as in Figure 1, “MLC”). Graphs depict means ± SEM of two independent experiments in addition to each replicate. Outliers were removed when permissible by ROUT method. **(A,B)** CD4^+^ and CD8^+^ T cell activation at 24 h was determined as CD69 positivity by flow cytometry. *n* = 8–10. *p < 0.05 compared to MLC control, #*p* < 0.05 compared to MLC d7 (one-way ANOVA with Sidak posttest. **(C,D)** IL-2 production was quantified at 24 h by cytometric bead assay. *n* = 8–10. *p < 0.05 compared to control (Student *t*-test). **(E,F)** CD4^+^ and CD8^+^ T cell activation at 48 h was determined as CD25 positivity by flow cytometry. *n* = 8–10. *p < 0.05 compared to MLC control, #*p* < 0.05 compared to MLC d7 (one-way ANOVA with Sidak posttest. **(G,H)** IFN-γ production was quantified at 48h by cytometric bead assay. *n* = 4–6. * *p* < 0.05 compared to control (Student *t-*test).

Given that the previous results were obtained from whole splenocyte cultures, it is unclear whether the observed defects constitute and intrinsic defect of the T cells, or if they are mediated by cell-to-cell contact with inhibitory cells and/or the release of soluble inhibitory factors by non-T cells. Initially, we examined spleen histology after scald injury and noted indications of extramedullary hematopoiesis in the red pulp, while the lymphocyte zones seemed intact (Figure S1A). This potentially indicated an extrinsic, cell-mediated mechanism. In line with this, we noted a negative correlation between T cell activation and spleen/body mass ratio in PBD7 samples (Figure S1B). To support this further, we purified T cells using anti-CD90.2 magnetic bead separation and compared T cell activation in these purified samples to the mixed lymphocyte cultures (MLC). Purification of T cells from PBD7 samples significantly improved CD4^+^ and CD8^+^ T cell activation at both 24 h and 48 h (Figure 3). To address the question of whether the extrinsic suppression of T cells after burn injury is contact dependent, or whether a soluble mediator is responsible, we incubated control splenocytes with PBD7 splenocytes, but separated the two by a cell-impermeable mesh. Control splenocytes maintained normal T cell activation in this setting (Figure S1C), arguing against an impairment due to soluble mediators.

### *In vivo* Depletion of Gr1^+^ Cells Mitigates Scald-Induced T Cell Dysfunction

**Figure 4:**
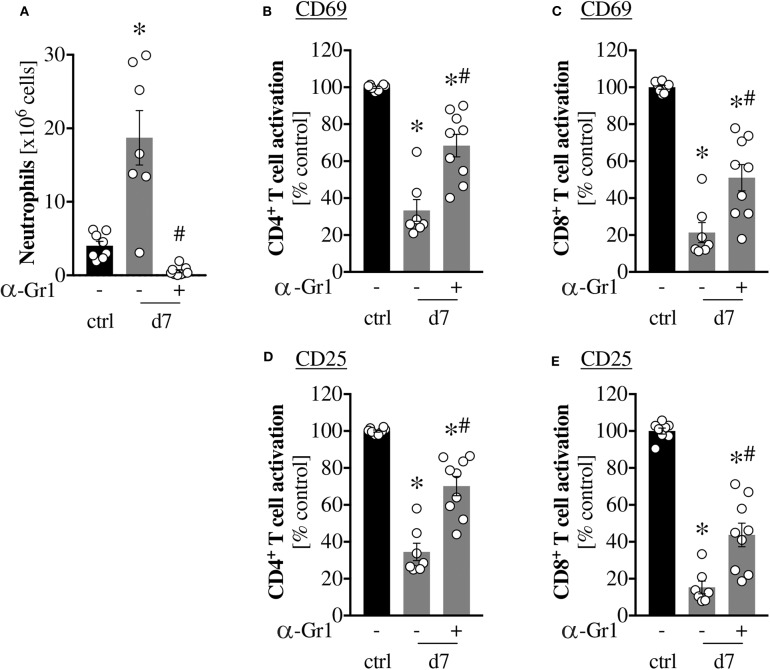
*in vivo* depletion of Gr1^+^ cells mitigates scald-induced T cell dysfunction. CD1 outbred mice were subjected to scald injury (28% TBSA, full thickness). Controls did not undergo the procedure (ctrl). When indicated, mice received an *i.p*. injection of 100 μg anti-Gr1 antibody (clone RB6-8C5) on day 6 after injury. Spleens were harvested the next day (d7). Neutrophil numbers were determined by flow cytometry **(A)** and splenocytes were cultured for 24 h **(B,C)** or 48 h **(D,E)** in the presence of anti-CD3/CD28 coated beads. T cell activation was determined by CD69 **(B,C)** and CD25 **(D,E)** positivity by flow cytometry. Graphs depict means ± SEM of two independent experiments in addition to each replicate. *n* = 8–9. Outliers were removed when permissible by ROUT method. *p < 0.05 compared to ctrl -, #*p* < 0.05 compared to d7 - (one-way ANOVA with Sidak posttest).

To determine which cell type mediates the observed T cell dysfunction, we depleted Gr1^+^ cells *in vivo* by injection of an anti-Gr1 antibody on PBD6 and assessed T cell activation the next day. Scald injured mice showed a significant increase in splenic neutrophils on PBD7, which was successfully depleted by anti-Gr1 antibody injection (Figure 4A). Gr1^+^ cell depletion significantly improved CD4^+^ and CD8^+^ T cell activation and both 24 h and 48 h (Figures 4B–E). A similar ameliorative effect on T cell activation was not observed upon neutrophil depletion using an anti-Ly6G specific antibody, despite successful reduction of splenic neutrophil numbers (Figure S2).

### Immune-Checkpoint Inhibitor Expressing Gr1^+^ Cells Increase and Infiltrate Germinal Centers After Burn Injury

**Figure 5:**
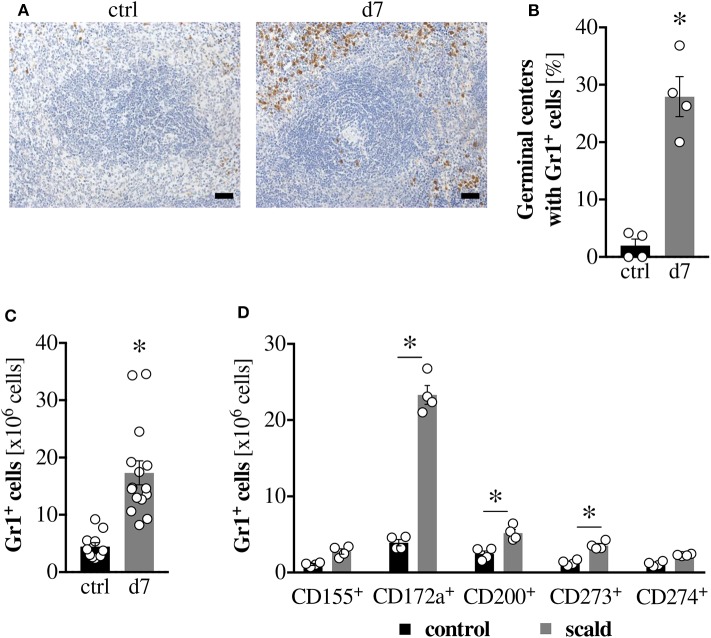
Immune-checkpoint inhibitor expressing Gr1^+^ cells increase and infiltrate germinal centers after burn injury. CD1 outbred mice were subjected to scald injury (28% TBSA, full thickness). Controls did not undergo the procedure (ctrl). Spleens were harvested on day 7 after injury (d7). Formalin-fixed, paraffin-embedded tissue sections were stained with an anti-Gr1 antibody and counterstained with hematoxylin. Scale bar indicates 1,000 μm. **(A)** Based on these sections, the percentage of germinal centers that show invasion of Gr1^+^ cells was determined by a blinded, experienced investigator. Four spleens per group and five entire sections per spleen were counted. Data points show the average percentage per individual spleen **(B)**. Numbers of total Gr1^+^ cells **(C)**, as well as the numbers of Gr1^+^ cells expressing various checkpoint inhibitors **(D)** were quantified in freshly isolated, unfixed spleens by flow cytometry. Graphs depict means ± SEM in addition to each replicate. *n* = 4 **(A,B,D)**, *n* = 12–15 **(C)**. Outliers were removed when permissible by ROUT method. *p < 0.05 compared to respective control [Student *t*-test **(B,C)** or one-way ANOVA with Sidak posttest **(D)**].

Given the amelioration of burn-induced T cell dysfunction upon Gr1^+^ cell depletion, we next investigated these cells in more detail. Histologically, PBD7 spleens showed an increase in Gr1^+^ cells (Figure 5A), which was confirmed by flow cytometry (Figure 5C). Importantly, histology showed an increase of Gr1^+^ cells into germinal centers in PBD7 samples, while they are normally confined to the red pulp (Figures 5A,B). This is of note because the germinal centers constitute the lymphocyte zones in the spleen, so the invading Gr1^+^ cells could be in direct contact with the splenic T cells. To assess how the Gr1^+^ cells induce T cell suppression, we quantified the numbers of checkpoint-inhibitor expressing Gr1^+^ cells by flow cytometry. The populations of CD172a, CD200 and CD273-expressing Gr1^+^ cells were significantly increased upon scald injury, with the CD172a^+^ subset showing the most pronounced increase (Figure 5D).

### *Ex vivo* Blockage of CD47, but Not CD172a Mitigates Scald-Induced T Cell Dysfunction

**Figure 6:**
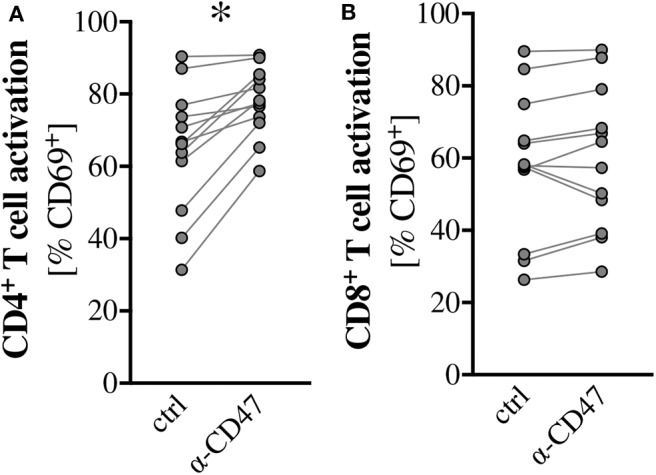
*Ex vivo* blockage of the CD172a ligand CD47 mitigates scald-induced T cell dysfunction. CD1 outbred mice were subjected to scald injury (28% TBSA, full thickness). Spleens were harvested on day 7 after injury and cultured for 24 h in the presence of anti-CD3/CD28 coated beads and with or without 2 μg/mL anti-CD47. CD4^+^ **(A)** and CD8^+^ **(B)** T cell activation was determined by CD69 positivity by flow cytometry. Graphs depict paired data from individual scald mice of two independent experiments. *n* = 12. *p < 0.05 compared to control (Wilcoxon signed-rank test).

Given the pronounced expansion of CD172a^+^ Gr1^+^ cells after burn injury and the successful restoration of T cell activation upon Gr1^+^ cell depletion, we wanted to assess if we could specifically block interactions of CD172a with its receptor, CD47, to ameliorate T cell dysfunction after burn. For this, we re-stimulated splenocytes *ex vivo* with anti-CD3/CD28 coated beads in the presence of anti-CD47. Blockage of CD172a did not improve CD4^+^ or CD8^+^ T cell activation (data not shown), but blockage of CD47 did significantly improve CD4^+^ T cell activation (Figure 6A), albeit not CD8^+^ T cell activation (Figure 6B).

### Scald Injury Leads To an Expansion of Immature Reticulocytes, Driving Checkpoint-Inhibitor Expression on Gr1^+^ Cells

Previous studies have established a role of immature reticulocytes in T cell suppression (44, 47, 49). In one tumor model, CD45^+^ Ter119^+^ CD71^+^ cells were identified as robust immunosuppressors, whose transcriptome strongly resembled that of myeloid-derived suppressor cells (52). The study did not investigate why these cells express both CD45 and Ter119, but one conceivable explanation is that myeloid-derived suppressor cells ingested the immature reticulocytes. Another interesting finding of the study is that the tumor-bearing mice were anemic despite the increase in immature reticulocytes, indicating a blockage of erythrocyte maturation. This would be in line with a clearance of the precursor cells by myeloid-derived suppressor cell ingestion as well. Given the observed T cell dysfunction after burn injury on top of signs of extramedullary hematopoiesis in our model (Figure S1A) and the knowledge that burn injury also frequently causes anemia (53), we hypothesized that there may be an expansion of immature reticulocytes in the spleens after burn injury. Ingestion of these cells by the expanding Gr1^+^ cell population could drive their immune checkpoint inhibitor expression similar to what is known after efferocytosis (33–36).

**Figure 7:**
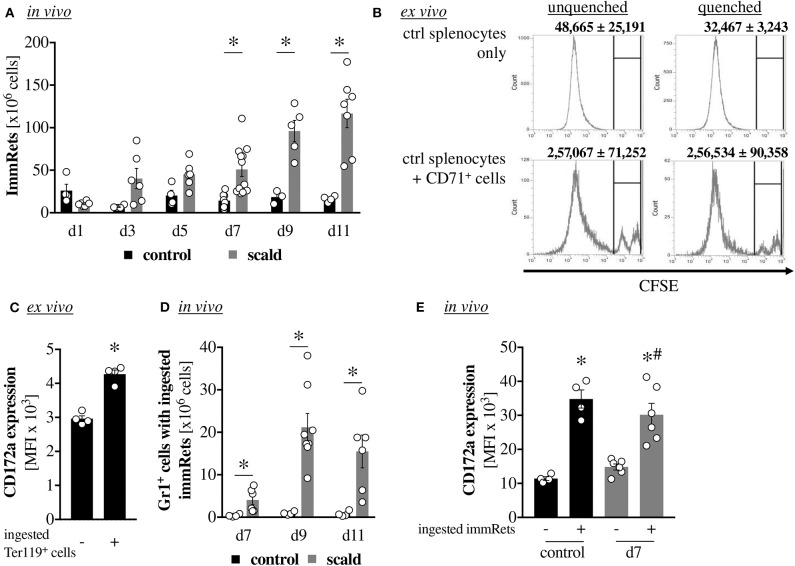
Scald injury leads to an expansion of immature reticulocytes, driving checkpoint-inhibitor expression on Gr1^+^ cells. CD1 outbred mice were subjected to scald injury (28% TBSA, full thickness). Controls did not undergo the procedure (ctrl). Spleens were harvested on the indicated days after injury. Numbers of immature reticulocytes (immRets, CD45^−^, Ter119^+^, CD71^+^) were quantified by flow cytometry. *n* = 4 (ctrl), *n* = 6–8 (scald) **(A)** To assess if these are ingested by myeloid cells, CD71^+^ cells were isolated from spleens after scald injury by jet-in-air cell sorting, labeled with CFSE and cultured overnight with unlabeled splenocytes obtained from control mice. The CFSE signal was quantified by flow cytometry in unquenched samples, as well as after quenching cell-surface signals with trypan blue. *n* = 4 **(B)** To determine if ingestion of immature reticulocytes drives CD172a expression on Gr1^+^ cells, Ter119^+^ cells were isolated by magnetic bead separation from spleens after scald injury and labeled with CFSE. Labeled cells were then incubated overnight with unlabeled splenocytes from control mice. CD172a expression on Gr1^+^ cells with and without ingestion of the labeled Ter119^+^ cells was determined by flow cytometry. *n* = 4 **(C)** *in vivo* numbers of Gr1^+^ cells with ingested immature reticulocytes was determined by intracellular Ter119 and CD71 staining and flow cytometry on the indicated days after burn **(D)** and expression of CD172a compared between Gr1^+^ populations with and without ingestion of immature reticulocytes **(E)** *n* = 4 (ctrl), *n* = 6–8 (scald). Graphs depict means ± SEM in addition to each replicate **(A,C–E)** or a representative histogram with means ± SEM **(B)** Outliers were removed when permissible by ROUT method. *p < 0.05 compared to respective control **(A,D)**, compared to – **(C)** or compared to control – **(E)**, #*p* < 0.05 compared to d7 – **(E)** [one-way ANOVA with Sidak posttest **(A,B,D,E)** or Student *t*-test **(C)**].

To test this, we first determined numbers of immature reticulocytes in spleens in a time course after burn injury. Immature reticulocytes were considered CD45^−^, Ter119^+^, and CD71^+^. We further confirmed the separation into mature and immature cells by determining CD49d expression and the presence of residual RNA and DNA by thiazole orange staining (Figure S3). We found an increase in immature reticulocytes, which became significant on PBD7 and continued until PBD11 (Figure 7A). To determine whether other splenocytes could ingest these cells, we incubated control splenocytes with CFSE-labeled CD71^+^ purified cells isolated from scald injured mice. Upon co-incubation, a CFSE-positive population of splenocytes was observed. Trypan blue addition did not quench the CFSE signal in these cells, indicating that the splenocytes ingested the CFSE-labeled CD71^+^ cells (Figure 7B).

To test our hypothesis that ingestion drives CD172a expression, we conducted a similar co-incubation with CFSE-labeled Ter119^+^ cells obtained from scald-injured mice. Gr1^+^ cells that ingested Ter11^+^ cells (positive for CFSE) showed higher expression of CD172a compared to those Gr1^+^ cells that did not (Figure 7C). To test whether similar ingestion occurs *in vivo*, we elucidated the numbers of Gr1^+^ cells with ingested immature reticulocytes based on intracellular CD71 and Ter119 staining after burn on those days that showed a significant increase in immature reticulocytes in the previous time course. For all three tested days (PBD7, PBD9, and PBD11), we noted a significant increase in Gr1^+^ cells which had ingested immature reticulocytes (Figure 7D). Compared to Gr1^+^ cells that did not ingest immature reticulocytes, those Gr1^+^ cells that did ingest them showed higher CD172a expression in both control and PBD7 cells (Figure 7E).

## Discussion

It's been previously established that burn injury causes T cell dysfunction. However, the duration of this dysfunction is unclear. In order to determine if T cell function is only impaired in the acute phase after burn or whether the changes persist long-term, we conducted a temporal study. We observed suppressed CD4^+^ and CD8^+^ T cell activation, as well as reduced IL-2 and IFN-γ production in the acute phase after burn, as well as up to 11 days after the injury. Neither activation nor cytokine production showed a clear indication of a beginning of recovery on PBD11. While there is no straight-forward algorithm to relate mouse days to human years, a general rule of thumb is that 9 days for a mouse constitute one human year (57). Thus, our data indicates that burn victims could demonstrate reduced T cell functionality for well over 1 year following the injury. This is in line with population studies, which found that burn patients exhibited a heightened risk for respiratory infections in the first five years following burn injury (58). Given that the leading cause of burn-related mortality are infections, this highlights the urgent need for clinical interventions (1).

The key to targeting burn-induced T cell dysfunction therapeutically is to understand the underlying mechanisms and identify potential interventions for mitigation. We first tested the hypothesis on whether the dysfunction is intrinsic to the T cells, or if it is instead mediated by an inhibitory cell. We noted enlarged spleens with evidence of extramedullary hematopoiesis in the red pulp after burn injury in our model, while the white pulp seemed inconspicuous. Additionally, higher spleen to body mass ratios in scald mice negatively correlated with T cell activation. Both indicated a potential cell-mediated defect. To test this, we assessed T cell activation in purified T cells and noted a significant improvement of functionality compared to mixed lymphocyte cultures. Further, control T cells maintained proper activation in the presence of mixed splenocytes obtained from scald mice, when the two populations were separated by a cell-impermeable mesh. This indicates that burn-induced T cell dysfunction is not mediated by a soluble mediator, but rather by a cell-to-cell contact dependent defect.

In order to identify the responsible inhibitory cell type, we depleted Gr1^+^ cells *in vivo* using an anti-Gr1 specific antibody. Gr1, a shared epitope of Ly6G and Ly6C surface antigens, is the murine marker for myeloid-derived suppressor cells (MDSCs). This cell type has known immunosuppressive functions and has been associated with disease severity, nosocomial infections and mortality in sepsis studies (59). Depletion of Gr1^+^ cells *in vivo* significantly ameliorated burn-induced T cell dysfunction in our model. This was specific to the depletion of Gr1^+^ cells, since neutrophil depletion with a Ly6G-specific antibody had no such effect. While the role of MDSCs in sepsis has been studied, little is known about this cell type with regard to burn injury, other than that these cells have been found to infiltrate the burn wound (60). Our study shows an expansion of splenic Gr1^+^ cells in response to burn injury, as well as an infiltration of Gr1^+^ cells into the germinal centers. In healthy controls, Gr1^+^ cells are confined to the red pulp. The infiltration into the germinal centers is noteworthy, since germinal centers are lymphocyte zones. Thus, the infiltrating Gr1^+^ cells could be in direct contact with splenic T cells *in vivo*, which, based on our previous data, is a prerequisite for burn-induced T cell suppression. Common cell-contact-dependent immunosuppressive mediators of MDSCs are checkpoint inhibitory receptors. We noted significant increases in Gr1^+^ cells expressing the checkpoint inhibitors CD200 (OX-2) and CD273 (PD-L2), but the expansion of CD172a expressing Gr1^+^ cells after burn injury was the most pronounced, with an approx. 6-fold increase.

In light of the increase of CD172a-expressing Gr1^+^ cells after burn, we wanted to assess if we could specifically block interactions of CD172a with its receptor, CD47, to ameliorate T cell dysfunction. *In vitro* use of an anti-CD172a antibody did not improve T cell activation, but anti-CD47 did significantly improve CD4^+^ T cell activation. It is possible that no beneficial effect with anti-CD172a was seen because not all surface receptors were sufficiently blocked due to the strong increase in CD172a expression. Also, the anti-CD172a antibody does not deplete the Gr1^+^ cells, so they are still present in the mixed splenocyte cultures and could circumvent the antibody blockage through receptor recycling, as well as suppress T cell functions via other checkpoint inhibitors. The observation that anti-CD47 treatment only ameliorated CD4^+^ T cell dysfunction could similarly be explained by differential receptor recycling between the CD4^+^ and CD8^+^ T cell populations. Another possibility is that T cell suppression by CD172a involves another ligand than the classical CD47 after burn injury. For instance, surfactant protein A and D (SP-A, SP-D) are also ligands for CD172a and have been attributed immunomodulatory roles, although they have not been reported to be expressed on T cells so far (61). Further studies are necessary to identify the mechanism underlying the restorative effect of anti-CD47 treatment on T cell activation. Given that our data show that T cells are intrinsically functional after burn injury, enabling the phagocytosis of dysfunctional T cells through blocking the “do not eat me” signal upon interaction of CD172a and CD47 seems unlikely. However, enabling phagocytosis of other suppressive or dysfunctional cells through blockage of their respective CD47 molecules may lead to cross-activation of T cells, thus restoring their function. Increasing T cell priming is hypothesized to underlie the success of CD47 blockade in tumor models (62). Additionally, anti-CD47 treatment may have a T cell-activating effect through CD47 itself: A previous study in Jurkat cells has shown that anti-CD47 in combination with anti-CD3 enhanced IL-2 production. This is thought to be due to anti-CD47 mediated interactions between CD47 and other plasma membrane molecules, resulting in CD47 providing a T cell co-stimulatory signal (63).

It is unclear what drives the expansion of CD172a-expressing Gr1^+^ cells after burn injury. A study on immunosuppression by immature reticulocytes in tumor-bearing mice noted an expansion of CD45^+^ Ter119^+^ CD71^+^ cells (52). Ter119 and CD71 mark immature reticulocytes, which do not, however, normally express CD45. Since the study also reported that the transcriptome of these triple-positive cells strongly resembled that of MDSCs, we wondered if these cells are, in fact, Gr1^+^ cells that have ingested immature reticulocytes. The phagocytosis of immature reticulocytes could potentially drive CD172a expression, similar to what has been reported for the expression of other checkpoint inhibitors after efferocytosis (33–36). While the presence of immature reticulocytes has previously been reported in burn patients (64), they have not been studied in detail yet. It is also unclear if they are present in experimental burn models. Thus, we first quantified immature reticulocyte numbers in a time-course after burn. Burn injury did indeed result in an increase of immature reticulocytes, which became significant on PBD7 and continued to rise until PBD11. We further confirmed that these cells can be ingested by Gr1^+^ cells and that CD172a expression is higher on Gr1^+^ cells that have ingested immature reticulocytes than those that have not. Our data thus identify checkpoint inhibitor expression in response to ingestion of immature reticulocytes as a new pathway mediating T cell dysfunction after burn injury. Potentially, immature reticulocytes could also contribute to T cell suppression after burn themselves, as they have been attributed T cell suppressive properties in several studies (44, 47, 49).

We did not see a significant increase in immature reticulocytes acutely after burn injury, however, whereas T cell functionality was already significantly reduced. This indicates that a separate mechanism is responsible for the early inhibition of T cell activation. Our previous work demonstrates that T cells undergo apoptosis in the acute phase after burn (54), which may play a role. Further studies are necessary to distinguish between mediators of acute and persistent T cell dysfunction after burn injury.

Our data identify MDSCs and immature reticulocytes as new mediators in T cell suppression and thus as potentially novel clinical targets. Further studies are needed to determine what drives the expansion of these two populations. In the future, this could enable the development of new, urgently needed therapies to reduce long-term infection susceptibility after burn and lower infection-related morbidity and mortality in burn victims, which is unfortunately still the number one cause of postburn mortality.

## Data Availability Statement

The datasets generated for this study are available on request to the corresponding author.

## Ethics Statement

The animal study was reviewed and approved by Institution of Animal Care and Use Committee of the University of Cincinnati (IACUC number 08-09-19-01).

## Author Contributions

NB and FH: data collection, data analysis and interpretation, critical revision of the article, and final approval of the version to be published. NB, FH, MH, BP, VN, and CC: conception of the work, data analysis and interpretation, drafting the article, critical revision of the article, and final approval of the version to be published.

## Conflict of Interest

The authors declare that the research was conducted in the absence of any commercial or financial relationships that could be construed as a potential conflict of interest.
