# Supplementary figures and images for: Scald Injury-Induced T Cell Dysfunction Can Be Mitigated by Gr1+ Cell Depletion and Blockage of CD47/CD172a Signaling
Source: Front Immunol. 2020 May 8;11:876. doi: 10.3389/fimmu.2020.00876 (PMC7232553; doi:10.3389/fimmu.2020.00876)

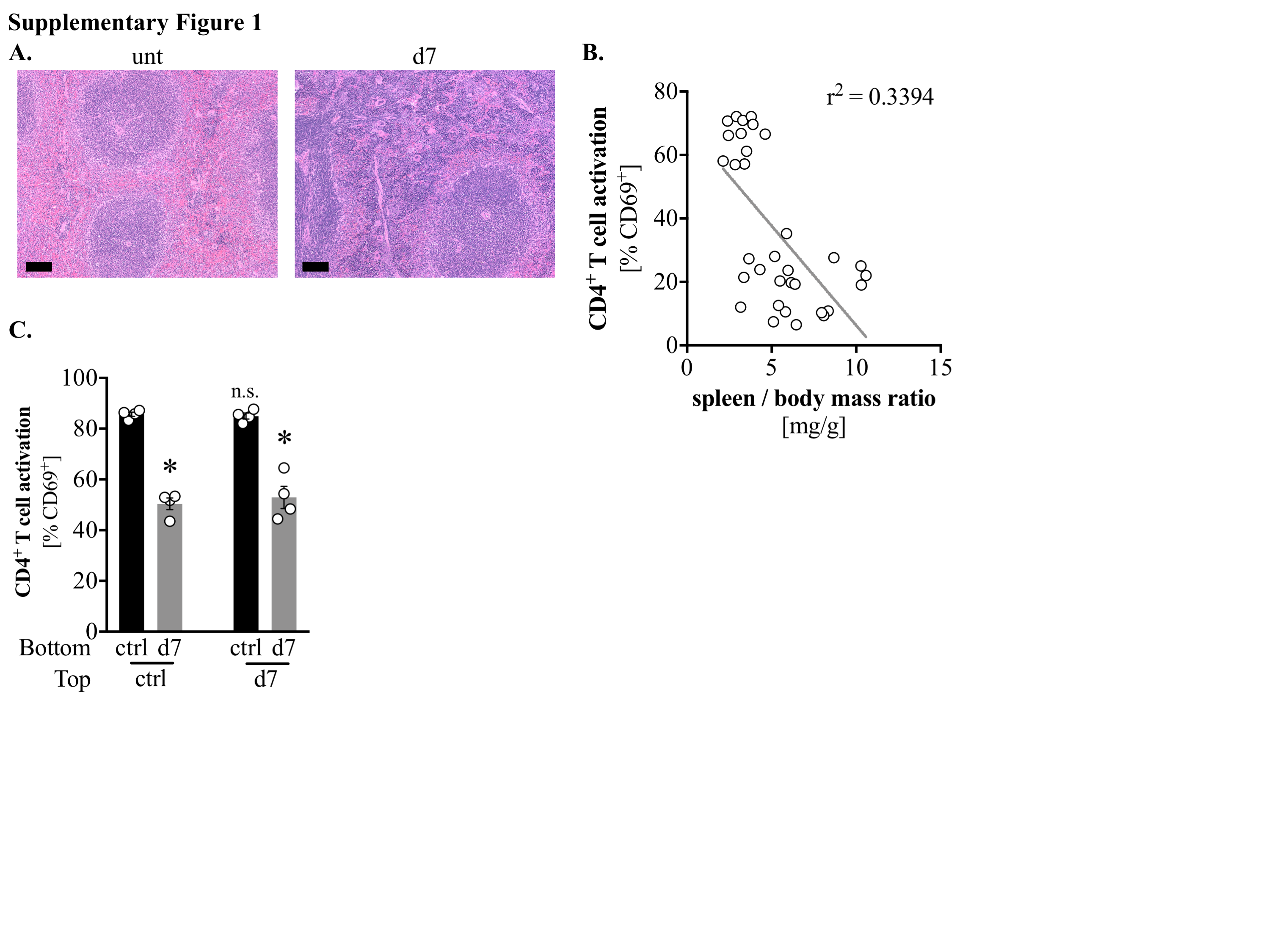

Supplement: Supplementary file 1 [file Image_1.TIFF]

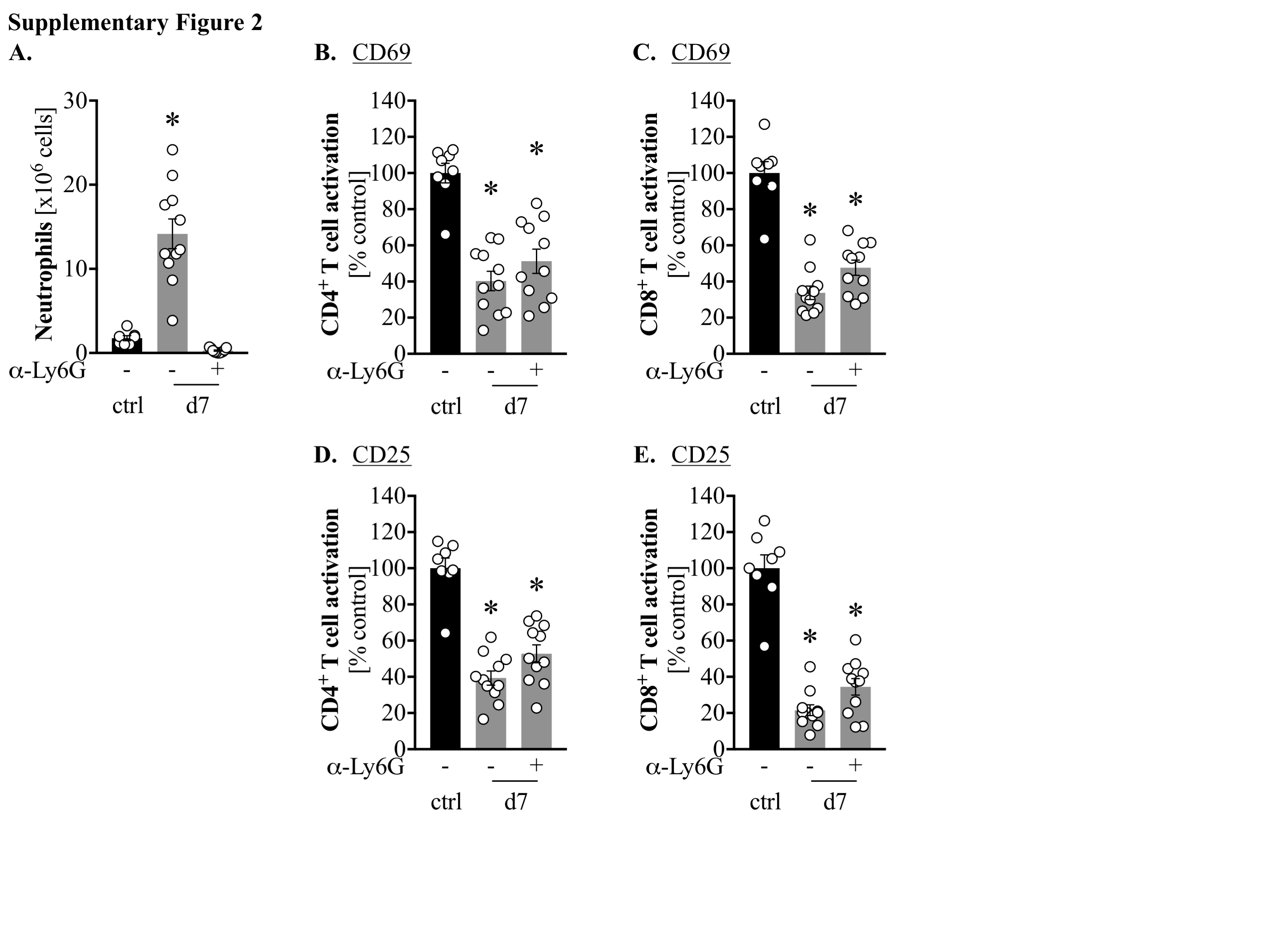

Supplement: Supplementary file 2 [file Image_2.TIFF]

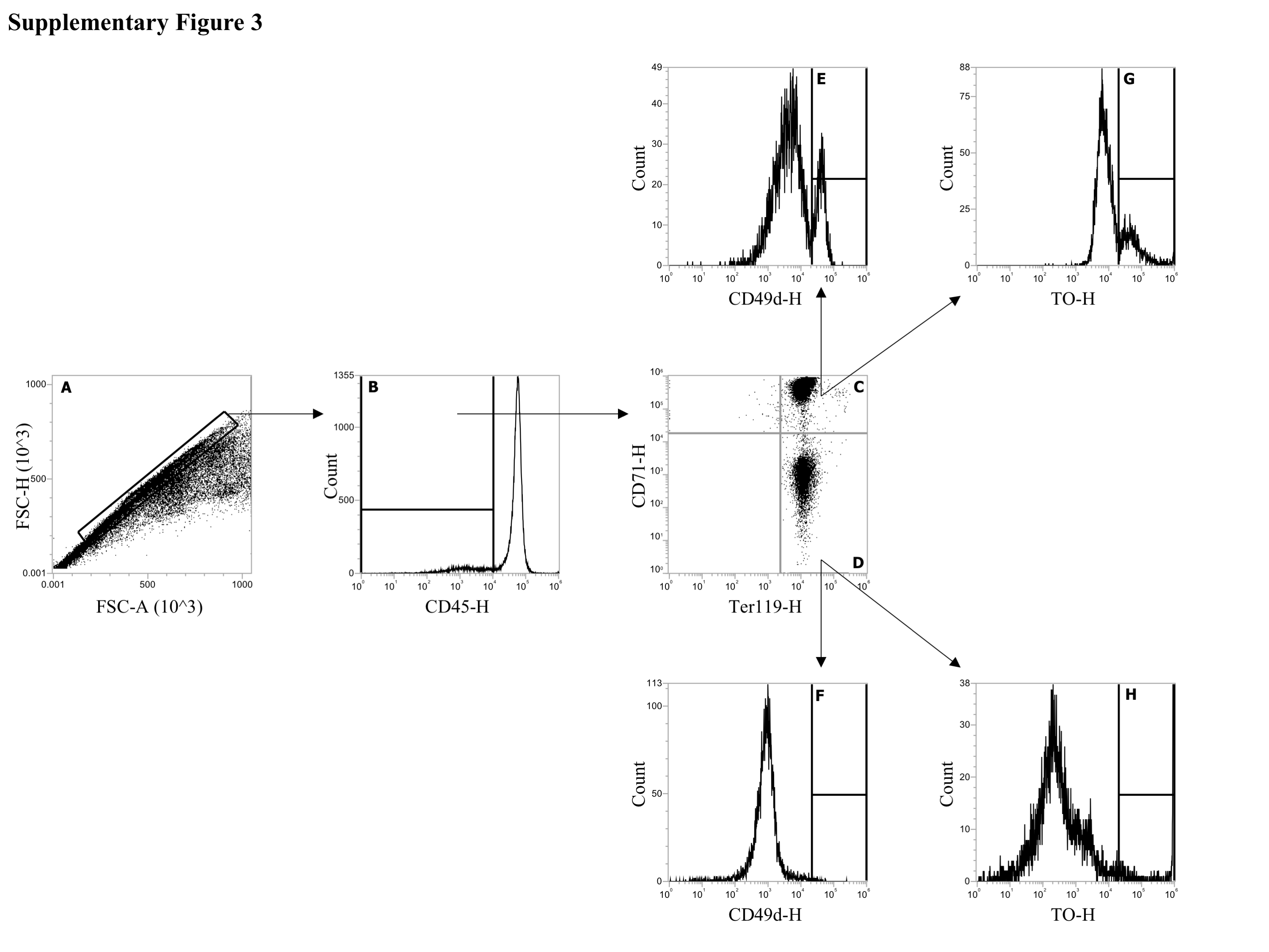

Supplement: Supplementary file 3 [file Image_3.TIFF]
