# Supplementary material for: Scald Injury-Induced T Cell Dysfunction Can Be Mitigated by Gr1+ Cell Depletion and Blockage of CD47/CD172a Signaling
Source: Front Immunol. 2020 May 8;11:876. doi: 10.3389/fimmu.2020.00876 (PMC7232553; doi:10.3389/fimmu.2020.00876)
Supplement: Supplementary file 4 [file Data_Sheet_1.docx]

Supplementary Material

**Supplementary Figure 1.** CD1 outbred mice were subjected to scald injury (28 % TBSA, full thickness). Controls did not undergo the procedure (ctrl). Spleens were harvested on day 7 after injury. (A) Formalin-fixed, paraffin-embedded tissue sections were stained with hematoxylin and eosin. n = 4. Scale bar indicates 100 µm. (B) Body- and spleen weight of PBD7 mice were determined. Splenocytes were cultured the presence of anti-CD3/CD28 coated beads for 24h. CD4^+^ T cell activation was determined by CD69 positivity by flow cytometry and graphed versus the spleen to body mass ratio. n = 33. r^2^ was calculated using Pearson correlation coefficient. (C) Control or d7 splenocytes were added to the bottom well of transwell plates and stimulated with anti-CD3/CD28 coated beads. Either control or d7 splenocytes were added to the cell-impermeable insert (Top) of the plate without addition of stimulating beads. After 24h, cells from the bottom well were recovered and CD4^+^ T cell activation determined by CD69 positivity by flow cytometry. Graphs depict means ± SEM in addition to each replicate. n = 4. * p < 0.05 compared to bottom ctrl/top ctrl (one-way ANOVA with Sidak posttest).

**Supplementary Figure 2.** CD1 outbred mice were subjected to scald injury (28 % TBSA, full thickness). Controls did not undergo the procedure (ctrl). When indicated, mice received an *i.p.* injection of 100 µg anti-Ly6G antibody (clone 1α8) on day 6 after injury. Spleens were harvested the next day (d7). Neutrophil numbers were determined by flow cytometry (A) and splenocytes were cultured for 24h (B, C) or 48h (D, E) in the presence of anti-CD3/CD28 coated beads. T cell activation was determined by CD69 (B, C) and CD25 (D, E) positivity by flow cytometry. Graphs depict means ± SEM in addition to each replicate. n = 8-11. Outliers were removed when permissible by ROUT method. * p < 0.05 compared to control (one-way ANOVA with Sidak posttest).

**Supplementary Figure 3.** Gating strategy for immature reticulocytes. Doublets were excluded (A), followed by gating on CD45 negative cells (B). These were further grouped into Ter119^+^ CD71^+^ cells (immature reticulocytes, C) and Ter119^+^ CD71^-^ cells (D). CD49 expression (E, F) and thiazole orange staining (G, H) were compared on both subsets to confirm a more immature status of Ter119^+^ CD71^+^ cells.
